# GMDCSA-24: A dataset for human fall detection in videos

**DOI:** 10.1016/j.dib.2024.110892

**Published:** 2024-09-02

**Authors:** Ekram Alam, Abu Sufian, Paramartha Dutta, Marco Leo, Ibrahim A. Hameed

**Affiliations:** a Department of Computer Science, Gour Mahavidyalaya, Malda, West Bengal 732142, India; b National Research Council of Italy, Institute of Applied Sciences and Intelligent Systems, 73100 Lecce, Italy; c Department of Computer Science, University of Gour Banga, English Bazar, West Bengal 732103, India; d Department of Computer & System Sciences, Visva-Bharati, Santiniketan, West Bengal 731235, India; e Department of ICT and Natural Sciences, Norwegian University of Science and Technology, Trondheim, Norway

**Keywords:** Indoor fall detection, Remote elderly care, Video classification, Video dataset

## Abstract

The population of older adults (elders) is increasing at a breakneck pace worldwide. This surge presents a significant challenge in providing adequate care for elders due to the scarcity of human caregivers. Unintentional falls of humans are critical health issues, especially for elders. Detecting falls and providing assistance as early as possible is of utmost importance. Researchers worldwide have shown interest in designing a system to detect falls promptly especially by remote monitoring, enabling the timely provision of medical help. The dataset ‘GMDCSA-24′ has been created to support the researchers on this topic to develop models to detect falls and other activities. This dataset was generated in three different natural home setups, where Falls and Activities of Daily Living were performed by four subjects (actors). To bring the versatility, the recordings were done at different times and lighting conditions: during the day when there is ample light and at night when there is low light in addition, the subjects wear different sets of clothes in the dataset. The actions were captured using the low-cost 0.92 Megapixel webcam. The low-resolution video clips make it suitable for use in real-time systems with fewer resources without any compression or processing of the clips. Users can also use this dataset to check the robustness and generalizability of a system for false positives since many ADL clips involve complex activities that may be falsely detected as falls. These complex activities include sleeping, picking up an object from the ground, doing push-ups, etc. The dataset contains 81 falls and 79 ADL video clips performed by four subjects.

Specifications Table

**Table:** 

Subject	Computer Vision and Pattern Recognition
Specific subject area	Human Fall Detection in Indoor Videos
Type of data	Raw Video (mp4), Text files (csv)
Data collection	The video dataset clips were captured using the integrated webcam of the HP 348 G5 laptop. The details are given below: • Laptop: HP G5 348 (intel core i5 8th Gen) • Camera: HP G5 348 Web Camera, 720p, 0.92 Megapixel (MP), 30 FPS
Data source location	Department of Computer Science and Application, Gour Mahavidyalaya, Malda, India.
Data accessibility	Repository name: ekramalam/GMDCSA24-A-Dataset-for-Human-Fall-Detection-in-Videos: 2.1 Data identification number: 10.5281/zenodo.13354453 Direct URL to data: https://doi.org/10.5281/zenodo.13354453
Related research article	Alam, E., Sufian, A., Dutta, P., & Leo, M. (2023). Real-Time human fall detection using a lightweight pose estimation technique. In International Conference on Computational Intelligence in Communications and Business Analytics (pp. 30–40). Cham: Springer Nature Switzerland.

## 1 Value of the Data

• Human falls, a significant health concern especially for elders, require early detection, emphasizing the importance of an efficient and accurate fall detection system [1,2]. So, researchers are increasingly interested in developing and implementing an efficient and accurate human fall detection system. This dataset can be used to train or test a human fall detection system.
• The video recording in the proposed dataset was captured in three different homes and multiple environments, including varying lighting conditions, by four subjects wearing different attires.
• Besides falls, the dataset contains many activities of daily living (ADL). Therefore, it also can be used for human activity recognition (HAR) [3,4].
• The dataset was generated using a low-resolution (0.92 MP) webcam, making it computationally efficient without further compressing or processing. That is, this raw data is suitable for real-time use on low-computing devices [1,5,6].
• Different occlusions are incorporated into the dataset to enrich its diversity.
• Some ADL videos in this dataset feature activities similar to falls, such as sleeping, doing push-ups, etc. These activities closely resemble falls. So, one of the goals of this dataset is to assess the robustness of fall detection systems in handling false positives.

## 2 Background

According to a report by the United Nations Population Fund (UNFPA), the average lifespan has increased globally from 45.51 years in 1950 to 73.16 years in 2023 [7]. Simultaneously, the average fertility rate has decreased from 5 in 1950 to 2.3 in 2021 on a global scale [7]. This is causing an imbalance where there are more aged persons than younger people, so support for the elders has to be largely increased, especially for their independent living. Falling is a common but potentially devastating experience for elders. If immediate medical care is not provided, it can be fatal or lead to disability [8]. Therefore, there is a need for indoor automated systems that can monitor and detect falls in elders in their homes. In this data article, we present a human fall dataset named ‘GMDCSA-24′ to assist researchers in developing models for detecting human falls and other non-fall activities (ADL). Some highlights of the released dataset are represented below.

• Though there are many human fall datasets [[9], [10], [11], [12], [13], [14], [15], [16], [17]], not all of them are easily accessible [[11], [14], [16]].
• The recording of many fall datasets has been done in a single home environment [12,13,15,18], unlike the proposed dataset, which has been recorded in three natural home environments. This diversity in environments provides a broader range of scenarios and variations, which can improve the robustness and generalizability of fall detection models trained on this dataset, leading to more accurate and reliable predictions in real-world applications.
• Many fall datasets [[10], [11], [12],[14], [15], [16], [17], [18]] do not include data with occlusions, unlike our proposed dataset, which contains many frames where subjects are occluded. Including occlusions provides a more realistic representation of real-world conditions, improving the model's ability to handle challenging scenarios and increasing the robustness and accuracy of the models.
• In one of the released datasets [9], only one actor is depicted, while in some datasets [16,17], the total number of subjects is not exactly provided by the dataset creators, unlike the proposed dataset, which includes four subjects. Having more subjects increases the diversity of the dataset, which helps fall detection models generalize better across different individuals. This diversity leads to improved model performance and reliability when applied to varied real-world scenarios.
• The total number of activities (fall and ADL) performed in many datasets is less than in the proposed dataset, and for some, it's not provided by the dataset creators [10,[12], [13], [14],^18^]. Having a greater number of activities increases the variety and complexity of the dataset, which helps machine learning models learn more comprehensive patterns. This leads to enhanced model accuracy and robustness when applied to diverse and complex real-world scenarios.
• The total storage size of the proposed dataset is also less than many available datasets [9,10,12,15,17,18]. A smaller storage size makes the dataset more manageable and accessible, facilitating faster data processing and reducing computational resource requirements. This can lead to quicker experimentation and model training, making it easier to deploy models in resource-constrained environments.
• Many existing datasets are of very high quality, making them unsuitable to use directly (without compressing or further processing) on low-resource computing devices.

Table 1 compares the proposed GMDCSA-24 dataset with the existing human fall dataset.

**Table 1:** Comparison of the existing vision-based human fall datasets based on size, accessibility, home environment, occlusion, No. of subjects, No. of videos, year, etc., with the proposed dataset.

Dataset Name	Sensor Type	Camera Type	Storage Size	Dataset Link	Accessible a	Multiple Home Setup	Occlusion	No. of Subj.	No. of ADLs	No. of Fall activities	Total Videos	Year
Le2i FDD [9]	Vision	RGB	17.44 GB	https://www.kaggle.com/datasets/tuyenldvn/falldataset-imvia	Yes	Yes	Yes	9	79	143	222	2013
URFD [10]	Vision	RGB, Depth	5.38 GB	http://fenix.ur.edu.pl/~mkepski/ds/uf.html	Yes	Yes	No	5	40	30	70	2014
SDUFall [11]	Vision	Depth		http://www.sucro.org/homepage/wanghaibo/SDUFall.html	No	–	No	10	1500	300	1800	2014
HQFSD [12]	Vision	WebCam	21.6	https://kuleuven.app.box.com/s/dyo66et36l2lqvl19i9i7p66761sy0s6	Yes	No	Yes	10	17	55	72	2016
TSFD [13]	Vision	Thermal	607 MB (zip)	https://drive.google.com/file/d/0ByBHFkIRDnx6S2M2WllKaVg5eGc/view?resourcekey=0-gK0m5-HyAqhpuwSNPYZOSQ	Yes	No	No	1	9	35	44	2016
SisFall [14]	Wearable, Vision	RGB		http://sistemic.udea.edu.co/en/research/projects/english-falls	No	–	No	15	19	15	34	2017
Up- fall Detection [15]	Wearable, Ambient, & Vision	RGB	850 GB	https://sites.google.com/up.edu.mx/har-up/	Yes	No	No	17	306	255	561	2019
E-FPDS [17]	Vision	RGB	2.42 GB^b^	https://gram.web.uah.es/data/datasets/fpds/index.html	Yes	Yes	No	Unknown	Image Dataset	Image Dataset	Image Dataset	2022
CAUCAFall [18]	Vision	RGB	7.75 GB	https://data.mendeley.com/datasets/7w7fccy7ky/4	Yes	No	No	10	50	50	100	2022
GMDCSA-24	Vision	RGB	1.95 GB	https://github.com/ekramalam/GMDCSA24-A-Dataset-for-Human-Fall-Detection-in-Videos	Yes	Yes	Yes	4	81	79	160	2024

a As of 21th July 2024.

b Including other supporting fillies.

## 3 Data Description

The ‘GMDCSA-24′ dataset is an extension of the ‘GMDCSA' dataset [1,5]. The original GMDCSA human fall dataset was created by performing fall and ADL activities with a single subject consisting of 16 fall and 16 ADL clips. The GMDCSA-24 dataset includes fall and ADL activities video clips using three additional actors in two new home setups. Recordings were made at different times of the day, with varying levels of lighting creating some challenges for model development in fall detection. As mentioned in the 'Value of the Data' section, the GMDCSA-24 dataset is unique in its use of a low-megapixel (0.92 MP) integrated laptop webcam, making it suitable to use directly (without any compression) on resource-constrained devices [19,20]. Another advantage of the GMDCSA-24 dataset is the inclusion of fall-like activities such as sleeping and doing push-ups, etc. in the ADL videos. This similarity between sleeping and falling poses a challenge for fall detection pipelines, as they may incorrectly classify sleep as a fall event. So, this dataset is valuable for testing the robustness of models in reducing false positives.

**Fig. 1:**
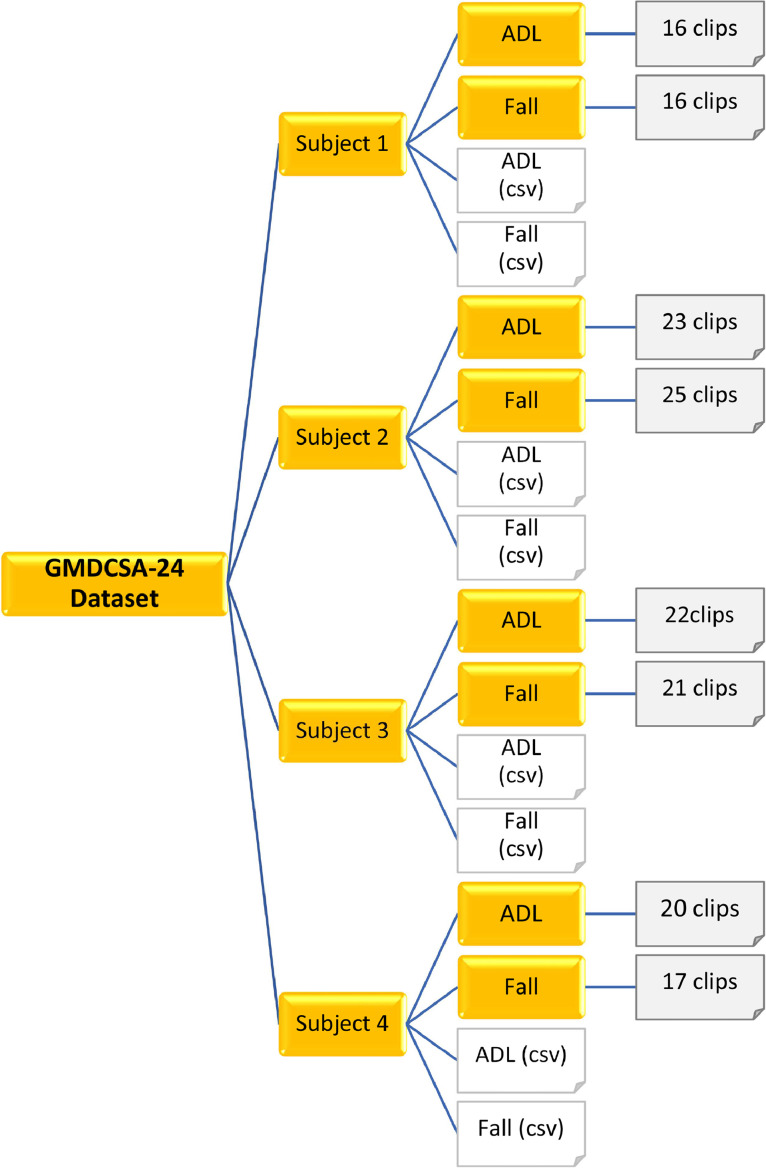
GMDCSA-24 dataset organizational structure.

Fig. 1 illustrates the organization of this dataset. The GMDCSA-24 dataset comprises four subdirectories: Subject 1, Subject 2, Subject 3, and Subject 4. Each directory, Subject 1, Subject 2, Subject 3, and Subject 4, contains two subdirectories: ADL and Fall, along with two CSV files, ADL.csv and Fall.csv. Each ADL and Fall directory contains video clips in MP4 format. Both the ADL and Fall directories under Subject 1 contain 16 video clips, resulting in a total of 32 video clips. Similarly, Subject 2 has 23 clips in the ADL directory and 25 clips in the Fall directory, while Subject 3 contains 22 ADL clips and 21 fall clips. Subject 4 contains 20 ADL clips and 17 fall clips. The basic details of the video clips of this dataset are shown in Table 2.

**Table 2:** Basic attributes of GMDCSA-24 dataset.

Attribute	Value
File type	mp4
Codec	H264
Frame Rate	30 fps
No. of Camera	1
No. of actors / Subjects	4
Total Storage Size	1.95 GB
No. of Home Environments	3

The CSV files describe the file name, length in seconds, time of recording, attire, description, and classes, as shown in Tables 3–and 5. The start and end times for each class are indicated in square brackets in seconds. To separate words within a field, a semicolon (';') is used instead of a comma (','), so it is not treated as a new field. The classes are listed in alphabetical order for ADL, and for falls, the fall classes are listed first in alphabetical order, followed by the ADL classes in alphabetical order. If a class appears multiple times in a clip, its timings are separated by a semicolon (';'), as shown in Table 4.

**Table 3:** Sample value of the ADL.csv file of the Subject 1 directory.

File Name,	Length (seconds),	Time of Recording,	Attire,	Description,	Classes
01.mp4,	08,	Day (Light On),	Full T-shirt & Trousers,	Sitting on the bed to sleeping right side on the bed. Face towards the camera,	Sitting[0 to 1]; Sleeping[2 to 8]

**Table 4:** Sample value of the ADL.csv file of the Subject 3 directory where there are multiple timings for a class.

File Name,	Length (seconds),	Time of Recording,	Attire,	Description,	Classes
04.mp4,	10,	Day,	Full T-shirt & Trousers,	Picking a book from the floor and putting it on the bed; sitting on the bed	Sitting[9.2 to 10]; Standing[2.4 to 3.6; 6.6 to 7.9; Walking[3.6 to 5.2; 7.9 to 9.2]

**Table 5:** Sample value of the Fall.csv file of the Subject 2 directory.

File Name,	Length (seconds),	Time of Recording,	Attire,	Description,	Classes
02.mp4,	12,	Night,	Full Shirt & Jeans,	Drinking water from the water bottle and then falling backward,	Falling (BW)[6.9 to 12]; Drinking[3.5 to 5.3]; Standing[3.6 to 6.9]; Walking[0 to 3.6]

The subject and class-wise details of the length and dimensions of the clips of this dataset are shown in Table 6. There are four subjects from Subject 1 to Subject 4. The length column displays the minimum, maximum, mode, median, and mean value of the clip duration in seconds. The Dimension column displays the two types of dimensions of the video clips of this dataset, along with the number of clips for each dimension.

**Table 6:** Attributes of the ADL and Fall videos.

Subject	Class	Length (seconds)					Dimension (No of clips)	
		Min	Max	Mode	Median	Mean	1280 × 720	640 × 480
Subject 1	ADL	3	12	6	6	6.5	16	0
	Fall	4	6	6	5	5.06	13	3
Subject 2	ADL	4	13	11	11	10	22	1
	Fall	4	15	11	7	8.04	22	3
Subject 3	ADL	3	12	8	9	8.5	19	3
	Fall	3	12	6	6	6.52	19	2
Subject 4	ADL	4	17	12	8.5	9.25	17	3
	Fall	2	7	6	5	5.11	14	3

Table 7, Table 8 provide a brief description of the ADL and fall activities performed by Subject 2. In the same manner, Table 9, Table 10 describe the ADL and fall activities of Subject 3. Similarly, Table 11, Table 12 outline the ADL and fall activities of Subject 4.

**Table 7:** ADL class files descriptions of Subject 2.

File Name	Length (seconds)	Time of Recording	Attire	Description (Activities)
01.mp4	11	Day	Full Shirt & Pant	Talking on the mobile phone while sitting on the bed and walking.
02.mp4	11	Day	Full Shirt & Pant	Picking up a water bottle from the ground and drinking while sitting on the bed.
03.mp4	11	Day	Full Shirt & Pant	Walking, removing dust from the bed using a pillow, and sleeping on the bed.
04.mp4	07	Day	Full Shirt & Pant	Yoga: Standing forward bend (Uttanasana).
05.mp4	10	Day	Full Shirt & Pant	Throwing a book on the bed, standing up from the ground, walking, and opening the curtain.
06.mp4	10	Day	Full Shirt & Pant	Picking up the mobile phone from the ground, placing it on the bed, and sitting on the bed.
07.mp4	13	Day	Full Shirt & Pant	Exercising (Moving both hands up and down).
08.mp4	13	Day	Full Shirt & Pant	Exercising (Stretching thigh and trying to touch the ground: Pyramid Pose).
09.mp4	10	Day	Full Shirt & Paint	Standing up from a sitting position on the bed and then sitting on the ground.
10.mp4	07	Day	Full Shirt & Paint	Standing up from the ground and closing the curtain.
11.mp4	10	Day	Full Shirt, T-shirt & Pant	Removing the shirt and hanging it on the hanger.
12.mp4	07	Day	T-shirt and Pant	Going to bed and sleeping (belly facing downwards).
13.mp4	04	Day	T-shirt and Pant	Sitting on the ground and reading a book.
14.mp4	11	Night	Full Shirt & Jeans	Walking and reading something while sitting on the bed.
15.mp4	11	Night	Full Shirt & Jeans	Reading a newspaper while sitting on the bed and placing a pillow on the lap.
16.mp4	11	Night	Full Shirt & Jeans	Reading a newspaper, throwing the newspaper, and sleeping.
17.mp4	11	Night	Full Shirt & Jeans	Standing up from bed, walking, and checking the dress on the hanger.
18.mp4	11	Night	Full Shirt & Jeans	Walking, opening the window, and looking outside.
19.mp4	11	Night	Full Shirt & Jeans	Sitting on the ground and writing in a notebook.
20.mp4	11	Night	Full Shirt & Jeans	Standing up, picking up a water bottle, and drinking water while sitting on the ground.
21.mp4	10	Night	Full Shirt & Jeans	Picking up a pen from the ground and walking.
22.mp4	12	Night	Full Shirt & Jeans	Exercising (Push-up) and sitting.
23.mp4	07	Night	Full Shirt & Jeans	Eating biscuits while sitting on the bed with legs folded.

**Table 8:** Fall class files descriptions of Subject 2.

File Name	Length (seconds)	Time of Recording	Attire	Description (Activities)
01.mp4	06	Day	T-Shirt & Pants	Right side fall on the ground.
02.mp4	08	Day	T-Shirt & Pants	Right side fall on the ground after walking.
03.mp4	07	Day	T-Shirt & Pants	Forward fall on the bed.
04.mp4	10	Day	T-Shirt & Pants	Seeing outside the window then backward fall.
05.mp4	04	Day	T-Shirt & Pants	Left side fall (face hidden by hand).
06.mp4	06	Day	T-Shirt & Pants	Walking while seeing the mobile phone then fall backward (left hand not fully in the frame).
07.mp4	04	Day	Full Shirt & Pants	Fall (forward) after trying to pick the bottle from the ground.
08.mp4	06	Day	Full Shirt & Pants	Reading a newspaper in a sitting position on the ground and then falling backward.
09.mp4	05	Day	Full Shirt & Pants	Falling backward after walking.
10.mp4	11	Night	Full Shirt & Jeans	Right side fall then backward.
11.mp4	10	Night	Full Shirt & Jeans	Falling from bed to ground.
12.mp4	12	Night	Full Shirt & Jeans	Drinking water from the water bottle and then falling backward.
13.mp4	08	Night	Full Shirt & Jeans	Falling backward (side view).
14.mp4	11	Night	Full Shirt & Jeans	Falling backward on the bed.
15.mp4	11	Day	T-Shirt & Pants 2	Writing while sitting on the bed, then falling on the ground.
16.mp4	04	Day	T-Shirt & Pants 2	Walking then falling forward.
17.mp4	06	Day	T-Shirt & Pants 2	Walking then falling forward.
18.mp4	11	Day	T-Shirt & Pants 2	Picking a water bottle from the ground and drinking water from it then falling forward on the ground.
19.mp4	15	Day	T-Shirt & Pants 2	Eating in standing position then falling forward.
20.mp4	11	Day	T-Shirt & Pants 2	Eating in standing position then falling forward.
21.mp4	09	Day	T-Shirt & Pants 2	Standing then falling sideways.
22.mp4	05	Day	T-Shirt & Pants 2	Walking then falling forward.
23.mp4	07	Day	T-Shirt & Pants 2	Standing then falling sideways.
24.mp4	07	Day	T-Shirt & Pants 2	Standing from the bed and then falling sideways on the ground.
25.mp4	07	Day	T-Shirt & Pants 2	Standing then falling backward on the ground.

**Table 9:** ADL class files descriptions of Subject 3.

File Name	Length (second)	Time of Recording	Attire	Description (Activities)
01.mp4	08	Day	Full T-shirt & Trouser	Writing something in the notebook while sitting on the bed.
02.mp4	09	Day	Full T-shirt & Trouser	Exercise (Hand up & down).
03.mp4	09	Day	Full T-shirt & Trouser	Getting up from the sleeping position from the bed, Putting a mask on the face, walking.
04.mp4	10	Day	Full T-shirt & Trouser	Picking a book from the floor and putting it on the bed, sitting on the bed.
05.mp4	05	Day	Full T-shirt & Trouser	Exercise (hand down & up with body bending).
06.mp4	08	Day	Full T-shirt & Trouser	Doing exercise and then sitting down on the floor.
07.mp4	03	Day	Full T-shirt & Trouser	Doing Exercise (sitting and standing).
08.mp4	04	Day	Full T-shirt & Trouser	Sitting to sleeping on the floor.
09.mp4	07	Day	Full T-shirt & Trouser	Sleeping on the floor to sitting and then standing.
10.mp4	12	Day	Full shirt & Trouser	Opening the window curtain and the window.
11.mp4	12	Day	Full shirt & Trouser	Closing the window curtain and the window.
12.mp4	10	Night	Full T-shirt 2 & Trouser	Changing pages of a book while sitting on the bed, standing.
13.mp4	08	Night	Full T-shirt (different) & Trouser	Sitting on the floor.
14.mp4	08	Night	Full T-shirt (different) & Trouser	Sitting on the floor & drinking water from the water bottle.
15.mp4	07	Night	Full T-shirt (different) & Trouser	Reading a newspaper while sitting on the floor.
16.mp4	10	Day	Kurta & Trouser	Eating while sitting on the ground.
17.mp4	12	Night	Full T-shirt (different) & Trouser	Drinking water from a water bottle while sitting on the bed Putting the water bottle on the bed.
18.mp4	11	Night	Full T-shirt (different) & Trouser	Walking, sitting on the bed, and eating biscuits.
19.mp4	06	Night	Full T-shirt (different) & Trouser	Picking the pen from the floor and putting it on the bed.
20.mp4	09	Night	Full T-shirt (different) & Trouser	Talking on the phone while sitting on the bed.
21.mp4	10	Day	Kurta & Trouser	Drinking while sitting on the bed.
22.mp4	09	Day	Kurta & Trouser	Writing while sitting on the bed.

**Table 10:** Fall class files descriptions of Subject 3.

File Name	Length (seconds)	Time of Recording	Attire	Description (Activities)
01.mp4	03	Day	Full T-shirt & Trouser	Falling forward.
02.mp4	06	Day	Full T-shirt & Trouser	Falling on the bed (forward).
03.mp4	05	Day	Full T-shirt & Trouser	Falling backward on the floor.
04.mp4	05	Day	Full T-shirt & Trouser	Walking and then falling backward on the floor.
05.mp4	10	Day	Full T-shirt & Trouser	Falling slowly from standing position to the floor
06.mp4	05	Day	Full T-shirt & Trouser	Falling (backward) on the floor from the sitting position
07.mp4	09	Day	Full T-shirt & Trouser	Falling on the floor from the standing position.
08.mp4	06	Day	Full T-shirt & Trouser	Falling on the right side onto the floor from a standing position.
09.mp4	06	Day	Full T-shirt & Trouser	Reading the book while sitting on the ground, then falling backward.
10.mp4	04	Day	Full shirt & Trouser	Falling forward on the bed.
11.mp4	08	Day	Kurta & Trouser	Eating in standing position then falling forward.
12.mp4	04	Day	Full T-shirt & Trouser	Falling backward from the bed.
13.mp4	04	Day	Full T-shirt & Trouser	Writing something in a notebook while sitting position on the floor, then falling backward.
14.mp4	12	Day	Full T-shirt, Shirt & Trouser	Falling backward on the floor while removing the shirt and trying to hang it on the hangar.
15.mp4	05	Night	Full T-shirt (different) & Trouser	Trying to drink water from the water bottle and then falling on the floor.
16.mp4	06	Night	Full T-shirt (different) & Trouser	Walking and then falling left side on the floor.
17.mp4	08	Night	Full T-shirt (different) & Trouser	Reading newspaper then falling Backward on the bed.
18.mp4	11	Night	Full T-shirt (different) & Trouser	Sitting on the bed, then falling on the floor.
19.mp4	05	Night	Full T-shirt (different) & Trouser	Falling (Backward) on the floor from the standing position.
20.mp4	06	Night	Full T-shirt (different) & Trouser	Walking then falling forward on the floor.
21.mp4	09	Day	Kurta & Trouser	Drinking in standing position then falling sideways.

**Table 11:** ADL class files descriptions of Subject 4.

File Name	Length (seconds)	Time of Recording	Attire	Description (Activities)
01.mp4	12	Day (Light Off)	Full Shirt & Pant	Wiping the face with a handkerchief while sitting on the bed, cleaning the glasses, and putting them on.
02.mp4	10	Day (Light Off)	Full Shirt & Pant	Talking on the mobile phone while walking.
03.mp4	08	Day (Light Off)	Full Shirt & Pant	Reading a book while sitting on the bed.
04.mp4	13	Day (Light Off)	Full Shirt & Pant	Using a laptop while sitting on the bed.
05.mp4	07	Day (Light Off)	Full Shirt & Pant	Doing exercise (push-ups), view 1.
06.mp4	06	Day (Light Off)	Full Shirt & Pant	Doing exercise (push-ups); view 2.
07.mp4	04	Day (Light Off)	Full Shirt & Pant	Doing exercise (push-ups), view 3.
08.mp4	12	Day (Light Off)	Full Shirt & Pant	Sleeping to sitting; putting on the glasses and drinking water.
09.mp4	12	Day (Light Off)	Full Shirt & Pant	Reading a book and checking the mobile phone while sitting on the bed.
10.mp4	12	Day (Light Off)	Full Shirt & Pant	Walking and then sleeping on the bed after removing the glasses.
11.mp4	10	Day (Light Off)	Full Shirt & Pant	Copying content from a book into a notebook while sitting on the floor.
12.mp4	08	Day (Light Off)	Full Shirt & Pant	Stopping copying from a book and drinking water while sitting on the floor.
13.mp4	05	Day (Light On)	Full Shirt & Pant	Writing while sitting on the chair.
14.mp4	08	Day (Light On)	Full Shirt & Pant	Sitting in the chair to standing on the floor; then picking up the water bottle and drinking water from it.
15.mp4	05	Day (Light On)	Full Shirt & Pant	Sitting to sleeping on the bed.
16.mp4	07	Day (Light On)	Full Shirt & Pant	Sitting on the bed; removing the glasses; then sleeping.
17.mp4	06	Day (Light On)	Full Shirt & Pant	Picking up the book from the floor and putting it on the bed.
18.mp4	14	Day (Light On)	Full Shirt & Pant	Wiping the face with a handkerchief; putting on the glasses; then reading a book.
19.mp4	09	Day (Light On)	Full Shirt & Pant	Walking; then picking up the biscuit jar and eating biscuits from it after sitting on the bed.
20.mp4	17	Day (Light On)	Full Shirt & Pant	Eating biscuits then picking up the water bottle from the floor and drinking the water from it after sitting on the bed; keeping the bottle on the floor.

**Table 12:** Fall class files descriptions of Subject 4.

File Name	Length (seconds)	Time of Recording	Attire	Description (Activities)
01.mp4	04	Day (Light On)	Full Shirt & Pant	Standing on the bed to falling (LS) on the bed.
02.mp4	02	Day (Light On)	Full Shirt & Pant	Falling (LS) from the bed to the floor.
03.mp4	06	Day (Light Off)	Full Shirt & Pant	Walking then falling (forward) view 1.
04.mp4	04	Day (Light Off)	Full Shirt & Pant	Walking then falling (forward) view 2.
05.mp4	07	Day (Light Off)	Full Shirt & Pant	Falling (RS) on the bed from sitting on the bed.
06.mp4	06	Day (Light Off)	Full Shirt & Pant	Falling (LS) on the bed from sitting on the bed and then falling to the floor.
07.mp4	06	Day (Light Off)	Full Shirt & Pant	Walking then falling (LS) on the floor.
08.mp4	06	Day (Light Off)	Full Shirt & Pant	Falling backward on the bed from sitting on the bed.
09.mp4	06	Day (Light On)	Full Shirt & Pant	Falling backward on the bed from sitting on the bed.
10.mp4	05	Day (Light On)	Full Shirt & Pant	Reading a book while sitting on the chair and then falling (FW) on the ground.
11.mp4	04	Day (Light On)	Full Shirt & Pant	Walking then falling (FW: only waist to toe is visible after falling).
12.mp4	04	Day (Light On)	T-Shirt & Pant	Falling (FW) to the floor from sitting on the bed.
13.mp4	05	Day (Light On)	T-Shirt & Pant	Falling (RS) on the bed from sitting on the bed.
14.mp4	05	Day (Light On)	T-Shirt & Pant	Falling (RS) on the bed from sitting on the bed then falling (LS) to the floor.
15.mp4	04	Day (Light On)	T-Shirt & Pant	Falling (FW) on the floor from sitting on the bed.
16.mp4	07	Day (Light On)	T-Shirt & Pant	Walking then falling (FW) on the floor.
17.mp4	06	Day (Light On)	T-Shirt & Pant	Falling (LS) on the bed from sitting on the bed.

Table 13, Table 14, Table 15, Table 16, Table 17, Table 18, Table 19, Table 20 list the individual activities that appear in each file in the Subject 1, Subject 2, Subject 3, and Subject 4 directories. This information can be useful for the task of HAR. We have only mentioned common activities like drinking, eating, exercising, reading, sitting, sleeping, standing, walking, and writing for the ADL class. Fall backward (BW), fall forward (FW), and fall sideways (SW) are also mentioned for the Fall class.

**Table 13:** File wise activities details of the ADL clips of Subject 1.

File Name	Reading	Sitting	Sleeping	Standing	Walking
01.mp4		✓	✓		
02.mp4		✓	✓		
03.mp4		✓	✓		
04.mp4		✓	✓		
05.mp4	✓	✓			✓
06.mp4	✓	✓			
07.mp4	✓	✓			
08.mp4					✓
09.mp4	✓				✓
10.mp4	✓				✓
11.mp4	✓	✓			✓
12.mp4	✓	✓		✓	✓
13.mp4		✓			✓
14.mp4		✓		✓	✓
15.mp4		✓			✓
16.mp4		✓			
**Total**	**7**	**13**	**4**	**2**	**9**

**Table 14:** File wise activities details of the Fall clips of Subject 1.

File Name	Falling (BW)	Falling (FW)	Falling (SW)	Sitting	Standing	Walking
01.mp4			✓	✓		
02.mp4			✓	✓		
03.mp4			✓	✓		
04.mp4			✓	✓		
05.mp4			✓			✓
06.mp4			✓			✓
07.mp4			✓			✓
08.mp4		✓				✓
09.mp4		✓			✓	
10.mp4		✓			✓	
11.mp4	✓				✓	
12.mp4	✓				✓	
13.mp4	✓				✓	
14.mp4	✓				✓	
15.mp4			✓	✓		
16.mp4			✓	✓		
**Total**	**4**	**3**	**9**	**6**	**6**	**4**

**Table 15:** File wise activities details of the ADL clips of Subject 2.

File Name	Drinking	Eating	Exercising	Reading	Sitting	Sleeping	Standing	Walking	Writing
01.mp4					✓		✓	✓	
02.mp4	✓				✓				
03.mp4					✓	✓		✓	
04.mp4			✓				✓		
05.mp4					✓		✓	✓	
06.mp4					✓		✓	✓	
07.mp4			✓				✓		
08.mp4			✓				✓		
09.mp4					✓		✓	✓	
10.mp4							✓	✓	
11.mp4							✓	✓	
12.mp4						✓	✓		
13.mp4				✓	✓		✓		
14.mp4				✓	✓			✓	
15.mp4				✓	✓				
16.mp4				✓		✓			
17.mp4							✓	✓	
18.mp4							✓	✓	
19.mp4					✓			✓	✓
20.mp4	✓				✓		✓		
21.mp4							✓	✓	
22.mp4			✓		✓				
23.mp4		✓			✓				
**Total**	**2**	**1**	**4**	**4**	**13**	**3**	**15**	**12**	**1**

**Table 16:** File wise activities details of the Fall clips of Subject 2.

File Name	Falling (BW)	Falling (FW)	Falling (SW)	Drinking	Eating	Reading	Sitting	Standing	Walking	Writing
01.mp4			✓					✓		
02.mp4			✓						✓	
03.mp4		✓								
04.mp4	✓							✓		
05.mp4			✓					✓		
06.mp4	✓								✓	
07.mp4		✓						✓		
08.mp4	✓					✓	✓			
09.mp4	✓								✓	
10.mp4	✓		✓						✓	
11.mp4	✓								✓	
12.mp4	✓			✓				✓	✓	
13.mp4	✓							✓		
14.mp4	✓							✓		
15.mp4			✓				✓			✓
16.mp4		✓							✓	
17.mp4		✓							✓	
18.mp4		✓		✓				✓	✓	
19.mp4		✓			✓			✓		
20.mp4		✓			✓			✓		
21.mp4			✓					✓	✓	
22.mp4		✓							✓	
23.mp4			✓					✓		
24.mp4			✓				✓	✓		
25.mp4	✓							✓		
**Total**	**10**	**8**	**8**	**2**	**2**	**1**	**3**	**14**	**11**	**1**

**Table 17:** File wise activities details of the ADL clips of Subject 3.

File Name	Drinking	Eating	Exercising	Reading	Sitting	Sleeping	Standing	Walking	Writing
01.mp4					✓				✓
02.mp4			✓				✓		
03.mp4					✓	✓	✓	✓	
04.mp4					✓		✓	✓	
05.mp4			✓				✓		
06.mp4			✓		✓				
07.mp4			✓				✓		
08.mp4					✓	✓			
09.mp4					✓	✓	✓		
10.mp4							✓		
11.mp4							✓	✓	
12.mp4				✓	✓		✓	✓	
13.mp4					✓				
14.mp4	✓				✓				
15.mp4				✓	✓				
16.mp4		✓			✓				
17.mp4	✓				✓				
18.mp4		✓			✓			✓	
19.mp4								✓	
20.mp4					✓				
21.mp4	✓				✓				
22.mp4					✓				✓
**Total**	**3**	**2**	**4**	**3**	**16**	**3**	**9**	**6**	**2**

**Table 18:** File wise activities details of the Fall clips of Subject 3.

File Name	Falling (BW)	Falling (FW)	Falling (SW)	Drinking	Eating	Reading	Sitting	Standing	Walking	Writing
01.mp4		✓								
02.mp4		✓						✓	✓	
03.mp4	✓							✓		
04.mp4	✓								✓	
05.mp4	✓							✓		
06.mp4	✓									
07.mp4	✓							✓		
08.mp4			✓							
09.mp4	✓					✓	✓			
10.mp4		✓								
11.mp4		✓			✓			✓		
12.mp4	✓							✓		
13.mp4	✓						✓			✓
14.mp4	✓							✓	✓	
15.mp4	✓							✓		
16.mp4			✓						✓	
17.mp4	✓					✓	✓			
18.mp4			✓				✓			
19.mp4	✓							✓		
20.mp4		✓							✓	
21.mp4			✓	✓						
**Total**	**12**	**5**	**4**	**1**	**1**	**2**	**4**	**9**	**5**	**1**

**Table 19:** File wise activities details of the ADL clips of Subject 4.

File Name	Drinking	Eating	Exercising	Reading	Sitting	Sleeping	Standing	Walking	Writing
01.mp4					✓				
02.mp4								✓	
03.mp4				✓	✓				
04.mp4					✓				
05.mp4			✓					✓	
06.mp4			✓					✓	
07.mp4			✓						
08.mp4	✓				✓	✓			
09.mp4				✓	✓				
10.mp4						✓		✓	
11.mp4					✓				✓
12.mp4	✓			✓	✓				✓
13.mp4					✓				✓
14.mp4	✓						✓	✓	
15.mp4					✓	✓			
16.mp4					✓	✓			
17.mp4								✓	
18.mp4				✓	✓				
19.mp4		✓			✓		✓	✓	
20.mp4	✓	✓			✓		✓		
**Total**	**4**	**2**	**3**	**4**	**13**	**4**	**3**	**7**	**3**

**Table 20:** File wise activities details of the Fall clips of Subject 4.

File Name	Falling (BW)	Falling (FW)	Falling (SW)	Reading	Sitting	Sleeping	Standing	Walking
01.mp4			✓				✓	
02.mp4			✓			✓		
03.mp4		✓						✓
04.mp4		✓						
05.mp4			✓		✓			
06.mp4			✓		✓			
07.mp4			✓					✓
08.mp4	✓				✓			
09.mp4	✓				✓			
10.mp4		✓		✓	✓			
11.mp4		✓						✓
12.mp4		✓			✓			
13.mp4			✓					
14.mp4			✓		✓			
15.mp4		✓			✓			
16.mp4		✓						✓
17.mp4			✓		✓			
**Total**	**2**	**7**	**8**	**1**	**9**	**1**	**1**	**4**

Table 21 summarizes all the activities and their frequencies by the four subjects of the GMDCSA-24 dataset. The rows of Table 21 are ordered alphabetically, first by ADL and then by fall activities.

**Table 21:** Summary Table of Table 13 to Table 20 to display the activities and their frequency in the dataset.

Activities	ADL: Frequency				Fall: Frequency				Total
	Sub. 1	Sub. 2	Sub. 3	Sub. 4	Sub. 1	Sub. 2	Sub. 3	Sub. 4	
Drinking		2	3	4		2	1		12
Eating		1	2	2		2	1		8
Exercising		4	4	3					11
Reading	7	4	3	4		1	2	1	22
Sitting	13	13	16	13	6	3	4	9	77
Sleeping	4	3	3	4				1	15
Standing	2	15	9	3	6	14	9	1	59
Walking	9	12	6	7	4	11	5	4	58
Writing		1	2	3		1	1		8
Fall (BW)					4	10	12	**2**	28
Fall (FW)					3	8	5	7	23
Fall (SW)					9	8	4	8	29

**Fig. 2:**
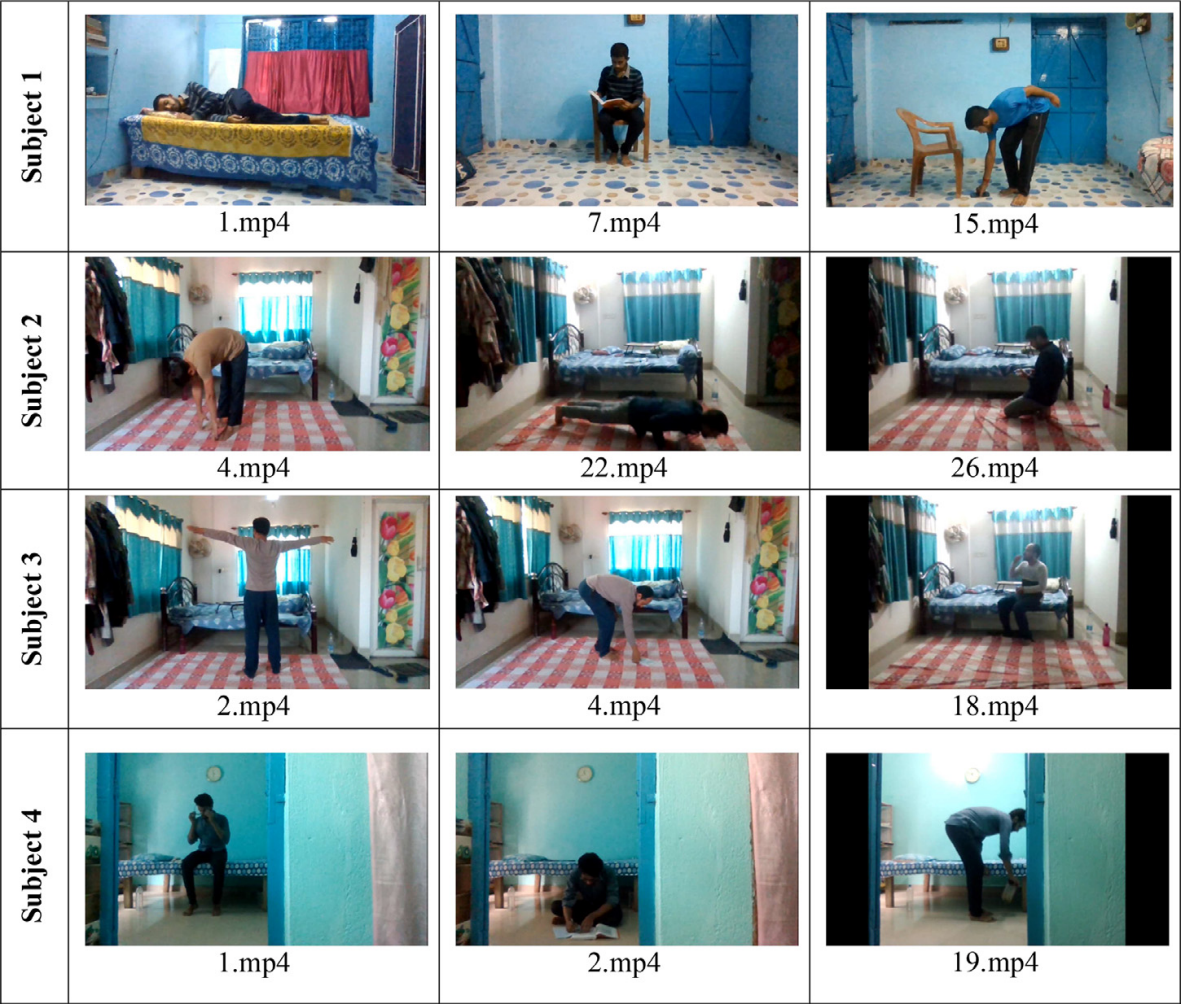
Some sample frames from the ADL class of the GMDCSA-24 dataset from all four subjects.

**Fig. 3:**
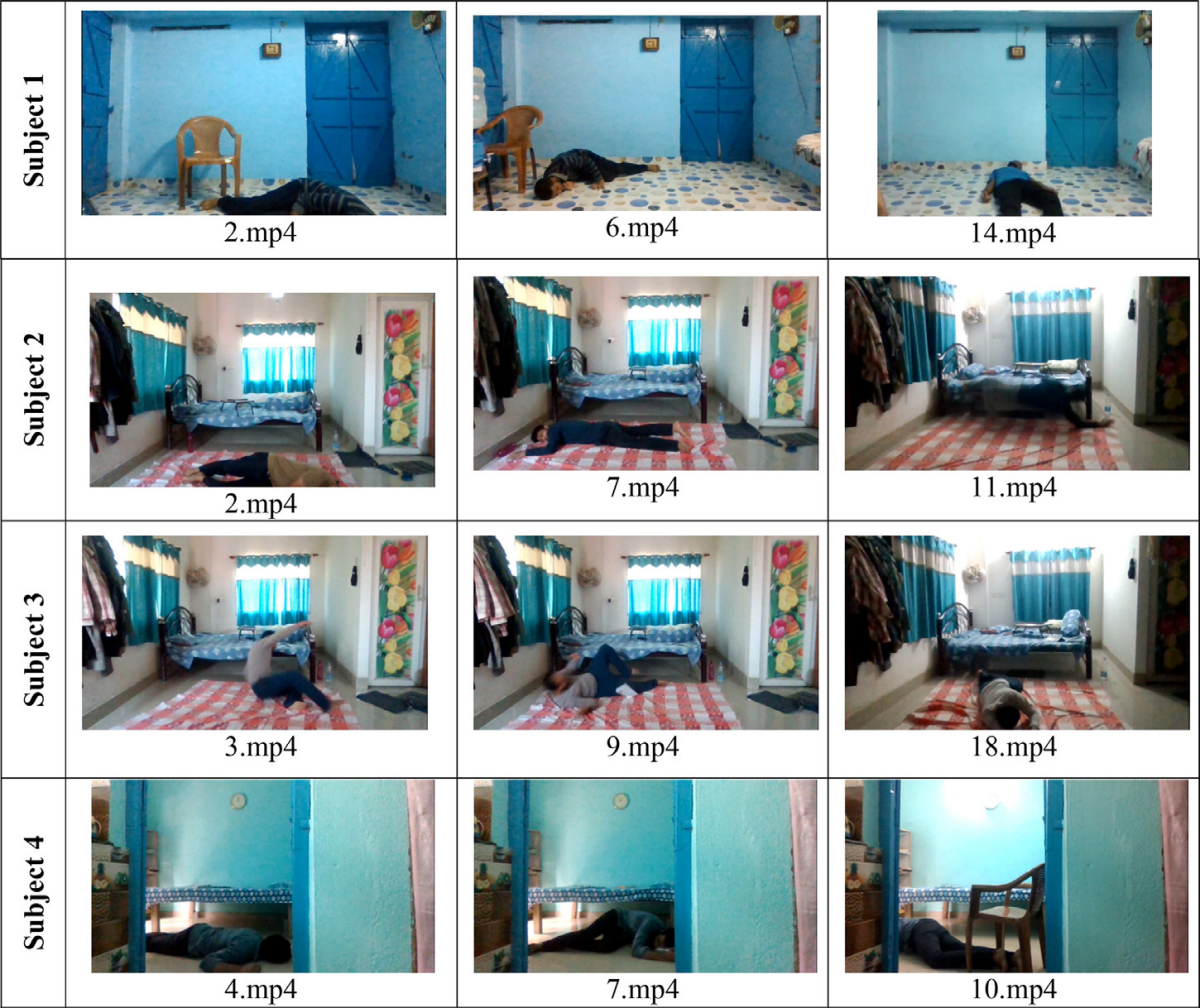
Some sample frames from the Fall class of the GMDCSA dataset from all four subjects.

Fig. 2, Fig. 3 show some sample frames from the ADL and fall video clips, respectively, from Subject 1, Subject 2, Subject 3, and Subject 4. The file names of each frame are shown below each image.

## 4 Experimental Design, Materials and Methods

This dataset was created by capturing the fall and ADL activities performed by four different subjects in three different home setups. The subjects were asked to perform random, natural, and common ADL and fall activities in indoor setups, wearing different sets of clothes and recording at different times of the day. This makes this dataset very suitable and versatile for any fall detection models. All subjects were informed about the use of this dataset, and consent was obtained from them. This dataset incorporates numerous ADL video sequences that closely resemble falls, featuring actions such as i) sleeping, ii) picking something up from the ground, iii) exercises similar to falls, like push-ups, etc. One of the primary uses of this dataset is to assess the system's robustness in detecting false positives. Additionally, the dataset boasts a lower resolution than other datasets, facilitating swift training and testing times without any further compression.

The camera (laptop) was kept fixed (static) while capturing the activities performed by all four subjects. As mentioned in the Data Description section, the video clips were captured using the 0.92 MP (720p, 30 FPS) webcam of the HP G5 348 laptop (Intel Core i5 8th Generation). The LosslessCut^1^ software was used to trim some lengthy video clips. The VLC media player^2^ was used to play back and check the videos. To record the videos, the Microsoft Camera (version 2024.2405.19.0) app^3^ was used. The classification of different ADL and fall activities was done manually after playing the video clips. The CSV files were prepared manually after playing the clips. The VLC extension, Time v3.2,^4^ was used to see the precise playback time.

Fall and ADL video clips were recorded in three natural room setups. Subject 1 performed in Room 1, Subject 2 and Subject 3 in Room 2, and Subject 4 in Room 3. The details of the rooms are provided in Table 22. Two camera positions were used in Room 1: one from the gate side towards the bed at a height of 70 cm and another from the bed towards the gate at a height of 90 cm. For Room 2 and Room 3, a fixed camera position (towards the bed) was used.

**Table 22:** Brief descriptions of the rooms used in this dataset.

Room	Subject	Room Width (cm)	Room Length (cm)	Camera Height (cm)	Bed Width (cm)	Bed Length (cm)	Bed Height (cm)
Room1	Subject 1	315	345	70, 90	135	185	50
Room 2	Subject 2, Subject 3	360	540	135	150	210	45
Room 3	Subject 4	180	282	64	135	185	50

## Ethics Statement

The data collection involved the participation of three human actors. Before recording video data, the participants were duly informed about the purpose of the data collection. All three were made aware of the intention to publish the dataset in a public repository. Among the three, two are the authors of this data article and one volunteer. All actors thoroughly read and signed an informed consent form. As per the authors' knowledge, ethics approval from an appropriate IRB/local ethics committee does not apply to this dataset.

## CRediT Author Statement

**Ekram Alam**: Conceptualization, Methodology, Investigation, Resources, Data curation, Writing – original draft, Writing – review & editing; **Abu Sufian**: Methodology, Investigation, Data curation, writing – review & editing, Supervision; **Paramartha Dutta**: Writing – review & editing, Supervision; **Marco Leo**: Writing – review & editing, Supervision, and **Ibrahim A. Hameed**: Writing – review & editing, Supervision.

## Data Availability

A dataset for human fall detection in videos (Original data) (Github).

## Footnotes

1 https://github.com/mifi/lossless-cut

2 https://www.videolan.org/vlc

3 https://apps.microsoft.com/detail/9wzdncrfjbbg

4 https://addons.videolan.org/p/1154032/
